# The Design and Development of an Injectable Thermoresponsive Hydrogel for Controlled Simvastatin Release in Bone Repair Applications

**DOI:** 10.3390/gels11120995

**Published:** 2025-12-10

**Authors:** Christopher R. Simpson, Helena M. Kelly, Ciara M. Murphy

**Affiliations:** 1 Tissue Engineering Research Group, Department of Anatomy & Regenerative Medicine, Royal College of Surgeons in Ireland (RCSI), 123 St. Stephen’s Green, D02 YN77 Dublin, Ireland; 2 School of Pharmacy and Biomolecular Sciences, Royal College of Surgeons in Ireland (RCSI), Ardilaun House, 111 St Stephen’s Green, D02 VN51 Dublin, Ireland; 3 Advanced Materials and Bioengineering Research (AMBER) Centre, Naughton Institute, Trinity College Dublin (TCD), D02 CP49 Dublin, Ireland; 4 Trinity Centre for Biomedical Engineering, Trinity College Dublin, 152-160 Pearse Street, D02 CP49 Dublin, Ireland

**Keywords:** hydrogel, biomaterials, controlled release, simvastatin, thermoresponsive, injectable

## Abstract

Osteoporotic vertebrae are a uniquely challenging tissue for local delivery due to the complex geometry of cancellous bone, the proximity of the spinal cord, and the need for reliable site retention. These challenges can be met with the use of stimuli responsive, state transiting formulations by leveraging their unique capacity for minimally invasive implantation as a liquid, sol–gel transition in response to stimuli, and finally, release of a loaded therapeutic. Here, we present the formulation development of a thermosensitive methylcellulose–collagen hydrogel, functionalised with controlled release simvastatin, recently shown to enhance osteogenesis while also impeding osteoclast activity. We first optimised a formulation with collagen content of 0.4% *w*/*v* to achieve a thermosensitive system with sol–gel transition at 29 °C, shear-thinning/injectable properties, low cytotoxicity, and high biocompatibility. Incorporation of nano-hydroxyapatite for enhanced bone tissue mimicry revealed optimal performance at 100% *w*/collagen content, showing long-term hydrolytic stability, maintaining more than 100% of its mass after 28 days. A loading concentration of 1 mg of simvastatin to 1 g of hydrogel displayed sustained release of simvastatin over 35 days. Finally, the release of simvastatin from the hydrogel into in vitro conditions prevented the formation of osteoclasts but failed to boost osteogenesis. Together these findings reveal a series of desirable stimuli-responsive hydrogel properties, achieving minimally invasive application coupled with sustained release of a hydrophobic compound, which is potentially useful for spatially complex bone regeneration. Further this work demonstrates the challenge of dosing sustained release systems to achieve simultaneous osteogenesis and anti-osteoclastogenic effects.

## 1. Introduction

Advances in biomaterial design have driven a shift toward minimally invasive solutions that promote healing while reducing surgical complications [1,2]. While implantable biomaterials remain widely used in bone regeneration, they often require invasive procedures and may struggle to conform to irregular defect sites, limiting their effectiveness in certain clinical applications. Certain bone defects, such as osteoporotic vertebral lesions, present challenges for traditional grafts and scaffolds due to difficulty with surgical access and irregular defect shapes. These challenges have led to increasing interest in injectable alternatives, which offer the ability to fill complex geometries while minimising surgical intervention [3]. Among these, hydrogels have garnered significant attention due to their tuneable properties, controlled therapeutic release, and ability to replicate key features of the native extracellular matrix [4].

Hydrogels are water swollen three-dimensional (3D) polymeric networks that are formed by inter- and intra-chain interaction of constituent polymers. They exhibit excellent biocompatibility and bio-resorption but further present an architecture and nutrient diffusion network that closely resembles native extracellular matrices [5]. Moreover, hydrogels have been shown as effective delivery platforms for water-soluble drugs and regenerative cells [6]. For injectable applications, hydrogels can possess in situ gelation properties, the ability to transition from a liquid to a solid gel state, a process known as sol–gel transition. A thermosensitive sol–gel is a property of physically crosslinking hydrogels designed to undergo state transition in response to physiological temperature. This property makes them suited for minimally invasive applications, as they remain free-flowing at room temperature before solidifying in situ, eliminating the need for chemical crosslinkers or external stimuli during clinical use [7]. These characteristics are advantageous for the repair of bone tissue with complex shapes at hard to access sites such as vertebral fragility fractures (VFFs) [8,9,10]. Moreover, their tunable viscosity and gelation time allow for precise control during application, enabling surgeons to achieve optimal placement and distribution.

Methylcellulose (MC), an abundant and biodegradable cellulose derivative, undergoes sol–gel transition at elevated temperatures of approximately 70 °C [11,12]. However, by incorporating salts such as β-glycerophosphate (β-GP), gelation temperatures can be reduced by a salting out effect, making MC suitable for sol–gel in biomedical applications [13,14]. Adding type I collagen addresses the lack of cell-binding sites in MC, as collagen is a major component of the native extracellular matrix and provides integrin-binding motifs such as the Glycine–Phenylalanine–Hydroxyproline–Glycine–Glutamic Acid–Arginine (GFOGER) sequence, which supports musculoskeletal cell adhesion [15,16,17]. Moreover, this combination provides complementary physical properties from each polymer, making a thermoresponsive system with potential as a clinically translatable delivery platform for stem cells and therapeutic molecules [18].

Hydroxyapatite (HAp) is the primary component of bone tissue, providing strength and rigidity to the skeletal system. Due to its excellent biocompatibility and its similarity to the mineral phase of human bones, when incorporated into porous polymer matrices, HAp offers cues that promote osteogenic differentiation and mineralization [19,20,21,22]. In addition, HAp enhances the physical attributes of polymer-based biomaterials [23]. Previous work in our research group reports the synergistic effects of HAp and collagen in scaffold design. Incorporating micron-sized HAp (mHAp) or nano-sized HAp (nHAp) into collagen scaffolds significantly improves their mechanical strength, surpassing that of collagen alone [24,25]. Moreover, collagen-HAp composite scaffolds have been shown to enhance the osteogenic effects on mesenchymal stem cells (MSC) differentiation compared to HAp-free scaffolds. This study combines MC, type I collagen, and HAp to leverage the discrete benefits of each material to develop a thermoresponsive biomimetic hydrogel suitable for minimally invasive delivery to bone.

The effectiveness of thermoresponsive hydrogels in bone repair depends on their ability to provide sustained release of bioactive molecules that promote osteogenesis (bone formation) and inhibit osteoclastogenesis (bone resorption). Given the high aqueous content of hydrogels, hydrophilic bioactive molecules are typically loaded due to compatibility and ease of release. In contrast, hydrophobic drugs are generally incompatible with the highly aqueous component of hydrogels. In these cases, hydrophobic drugs can have limited loading quantities and poor homogeneity in hydrogel matrices [26,27]. There are, however, approaches that confer hydrophobic properties to hydrogel polymers or leverage amphiphilic polymers to improve the loading compatibility of hydrophobic molecules [28].

Simvastatin (SVT) is a promising hydrophobic therapeutic for treating impaired bone remodelling. SVT is licenced to treat hyperlipidemia by inhibiting HMG-CoA reductase, but it has also shown promise in enhancing bone density in osteoporotic patients and animal models [29]. Subsequent studies involving direct stimulation of bone-related cells and tissue report supporting mechanisms of action [29,30,31,32]. These findings have translated to studies involving biomaterials loaded with statins and show a positive effect in regeneration of bone defects and osteogenic cultures [31,33,34]. The majority of these studies highlight the osteogenic effects of statins, but there is also evidence suggesting an anti-osteoclastogenic effect [35,36]. Despite these promising results, delivering sustained statin therapy to achieve simultaneous anabolic and anti-catabolic effects remains challenging because of their inherent hydrophobic nature.

This study details the development of a novel, thermoresponsive biomimetic MC/type I collagen/nHAp hydrogel designed to deliver a sustained release of SVT. This therapeutic hydrogel system was optimised to achieve key properties, including biocompatibility, injectability, thermoresponsive transition, resistance to degradation, and prolonged SVT release. While sustained SVT release was achieved, and the system inhibited osteoclast differentiation, it did not simultaneously promote bone formation. These findings show the challenges in sustained dosing to modulate both anabolic and catabolic processes using a single therapeutic modality. Nevertheless, this study demonstrates the potential of this system as a versatile platform for sustained delivery of hydrophobic therapeutics in spatially complex, minimally invasive bone regeneration applications.

## 2. Results and Discussion

Effective local bone regeneration and functional restoration remain critical challenges in treating bone defects, particularly in anatomically complex sites. Traditional solid biomaterials may struggle to conform to irregular defect geometries, whereas minimally invasive, injectable alternatives offer a promising solution by filling voids and transitioning in situ to provide structural support. Furthermore, therapeutic strategies for bone repair often require a dual approach—promoting bone formation while mitigating resorption. To meet these challenges in this study, we aimed to develop a temperature responsive MC/type I collagen/nHAp hydrogel designed to deliver a sustained release of SVT, hypothesised to have a synergistic effect on promoting bone formation and inhibiting bone resorption.

### 2.1. Rheological and Structural Analysis of MC Hydrogels with Increasing Collagen Content

**Figure 1:**
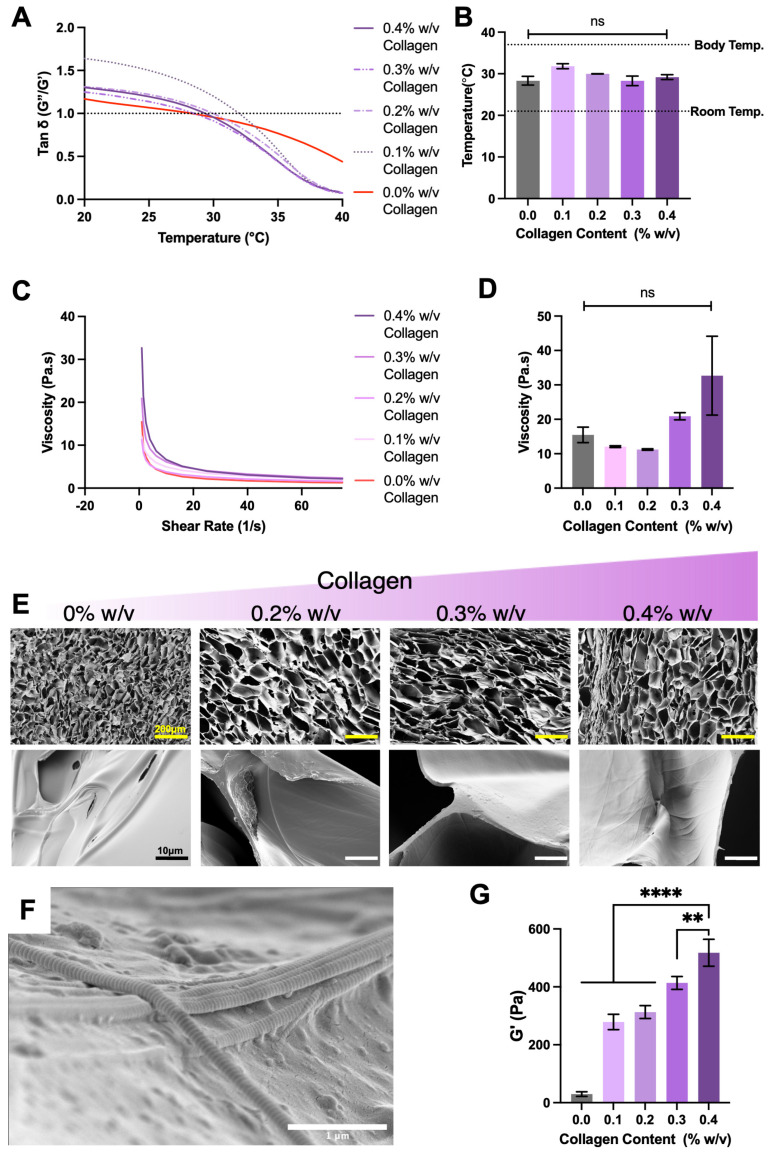
Rheological properties of modified collagen content formulations. (**A**) Ratio of storage and loss modulus as a function of temperature, as determined by oscillatory rheological assessment. Gelation was defined when G″/G′= 1. (**B**) Gelation temperature of different formulations. (**C**) Shear-thinning property at room temperature in all formulations, determined by rheological flow sweep. (**D**) Viscosity measured at a low shearing rate of 1.00 s^−1^. (**E**) SEM images of the microstructure of the hydrogels, scale bar top row = 200 μm, bottom row = 10 μm. (**F**) Confirmed collagen fibril presence with clear banding patterns visible on the surface. (**G**) Gelated elastic modulus determined by oscillatory rheological assessment after 30 min incubation at 37 °C. Data presented is the mean ± SD (*n* = 3) ns = not significant, ** = *p* < 0.01, **** = *p* < 0.0001.

**Figure A1:**
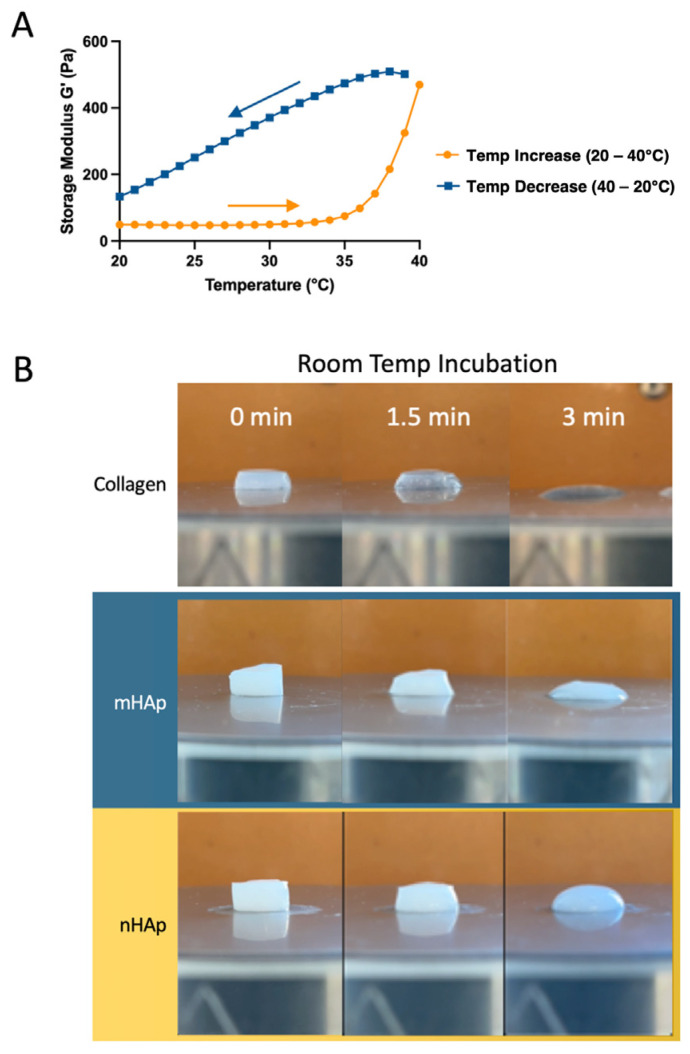
Gel-sol of nHAp-free, 200% mHAp and 100% nHAp. (**A**) Rheogram showing a reversal of storage modulus in response to lowering temperature. (**B**) Hydrogels formed into cylinders at 37 °C left at room temperature, deformed shape over time indicating transition from solid to liquid property.

Regenerative materials are typically designed to mimic the native extracellular matrix of the targeted tissue to maximise native cell interaction and subsequent tissue integration. These strategies, in some cases, can rely on the inherent bio-modulatory function of the materials. For this reason, the selection of type I collagen and optimisation testing was completed to present a collagen type associated with bone tissue. Minimally invasive characteristics were demonstrated by all formulations; this was specifically observed by all collagen concentrations undergoing sol–gel profiles and transition at temperatures ranging 28.3 °C and 31.8 °C (Figure 1A,B). The physical and temporary nature of MC crosslinking was confirmed by observing sol–gel reversal upon cooling (Appendix A Figure A1). These types of physical crosslink are described as dynamic reversible bonds elsewhere [37]. Further, all formulations showed shear-thinning profiles with increasing low shearing viscosities as collagen content increased (Figure 1C,D). Scanning electron microscopy revealed the influence on structural arrangement of methylcellulose in relation to collagen content, with collagen inclusion increasing hydrogel pore size (Figure 1E). Moreover, higher power magnification showed collagen fibrils closely associated with methylcellulose sheets (Figure 1F). Identification of collagen fibrils was confirmed by the presence of characteristic striped banding and fibril diameter reported in similar micrographs [38]. This interaction between collagen fibres and methylcellulose translated to a significant increase in viscoelasticity of gelated formulations as collagen content increases, with 0.4% collagen achieving a terminal elastic modulus of 517.8 ± 46.6 Pa, while methylcellulose alone reached 29.7 ± 4.6 Pa (Figure 1G).

The changes in viscoelasticity and low shear viscosity are likely due to a higher volume fraction of collagen fibres physically interacting with methylcellulose [39,40]. This higher degree of interaction would resist flow deformation and appear as greater elasticity. Despite these changes, the increased polymer interactions did not alter the sol–gel transition in any meaningful way; transition from liquid to solid occurred above room temperature but below body temperature, ideal for the intended injectable delivery strategy. Further, the low and identical viscosities at increasing shearing confirmed the ability of all formulations to thin out. Due to these properties the 0.4% *w*/*w* collagen formulation was taken to incorporate HAp.

### 2.2. Effect of Micro- and Nano-HAp on Rheological and Structural Properties of MC–Collagen Hydrogels

To further mimic bone ECM, many implantable biomaterial scaffolds for bone use ceramic inclusions. The mineral content has been shown to not only increase osteogenic property but also to reinforce the mechanical properties of the material. For example, nano-particulates such as graphene oxide, hydroxyapatite [41], and carbon nanotubes [42] have been used to enhance stiffness of various types of hydrogels or solid scaffolds. Increases in strength in this regard have been attributed to increased surface interaction within polymer chains or electrostatic charges enhancing interaction [41,43]. However, the effect of ceramics in composites is dependent on the intrinsic nature of the other composite components and can result in reductions in mechanical property, as reported with polylactic acid, for example.

**Figure 2:**
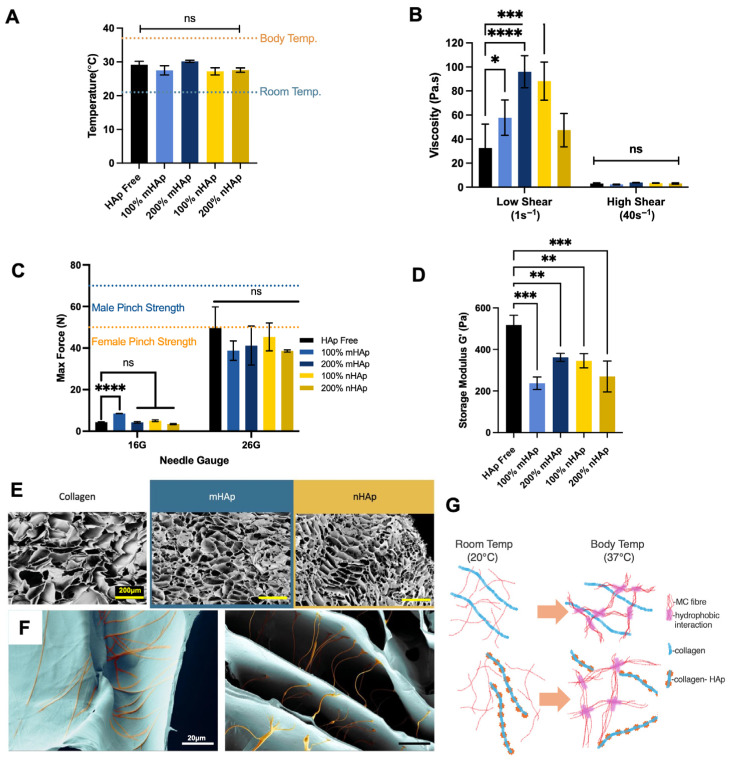
Rheological properties of hydroxyapatite content. (**A**) Gelation temperature with inclusion of nHAP and mHAp content. (**B**) Viscosity at low shears and high shearing rates of mHAp and nHAp. (**C**) Injection extrusion forces through a clinically relevant 16 G needle gauge were found to be low and were approaching the average female pinch strength when extruded via 26 G needles. (**D**) Rheological storage moduli were decreased by the inclusion of nHAp and mHAp. (**E**) Representative scanning electron microscopy images of mHAp, nHAp, and Hap-free formulations. (**F**) Higher magnification images showing collagen fibres (orange) associated with MC without HAp presence (**left**) and dissociating with nHAp (**right**). Scale bar = 200 μm top images, scale bar = 20 μm, bottom images. (**G**) Proposed schematic of changes in collagen interaction with methylcellulose with and without HAp. Diagram is not to scale. Data presented is the mean ± SD. (*n* = 3) ns = not significant, *p* ≤ 0.05 = *, *p* < 0.01 = **, *p* < 0.001 = ***, *p* < 0.0001 = ****.

To investigate the potential physical and osteogenic effects, nano- and micro-hydroxyapatite (nHAp and mHAp) were included into the 0.4% *w*/*w* collagen MC formulation at two concentrations. The inclusion of nHAp and mHAp did not significantly alter the sol–gel transition temperature compared to collagen alone, with the transition ranging from 27.2 °C to 30.2 °C (Figure 2A). Additionally, the hydrogel formulations with HAp maintained a shear-thinning property, reducing in viscosity with increasing shear rate. However, compared to Hap-free formulations there was a significant increase in viscosity with inclusion of 100% mHAp, 200% mHAp, and 100% nHAp at low shearing rates (Figure 2B). These differentials in viscosity were, however, lost at high shearing rates as the formulations thinned out. Importantly, HAp presence correlated with a decreased gelated elastic moduli 305.3 ± 82.5 Pa for mHAp 100% and 259.3 ± 57.7 Pa for nHAp 200% (Figure 2D). SEM images revealed that these changes in physical property were possibly due to dissociation of collagen fibres and with lower interaction with methylcellulose, observed by collagen fibres appearing in pores (Figure 2E,F). This change in collagen elastic behaviour, as a result of HAp inclusion, is proposed to not influence the thermoresponsive physical crosslinking but the association of collagen fibres and with MC (Figure 2G). Despite this, injectability remained unaffected, with all formulations extruding below the average human pinch strength, supporting feasibility of injectable delivery (Figure 2C).

**Figure 3:**
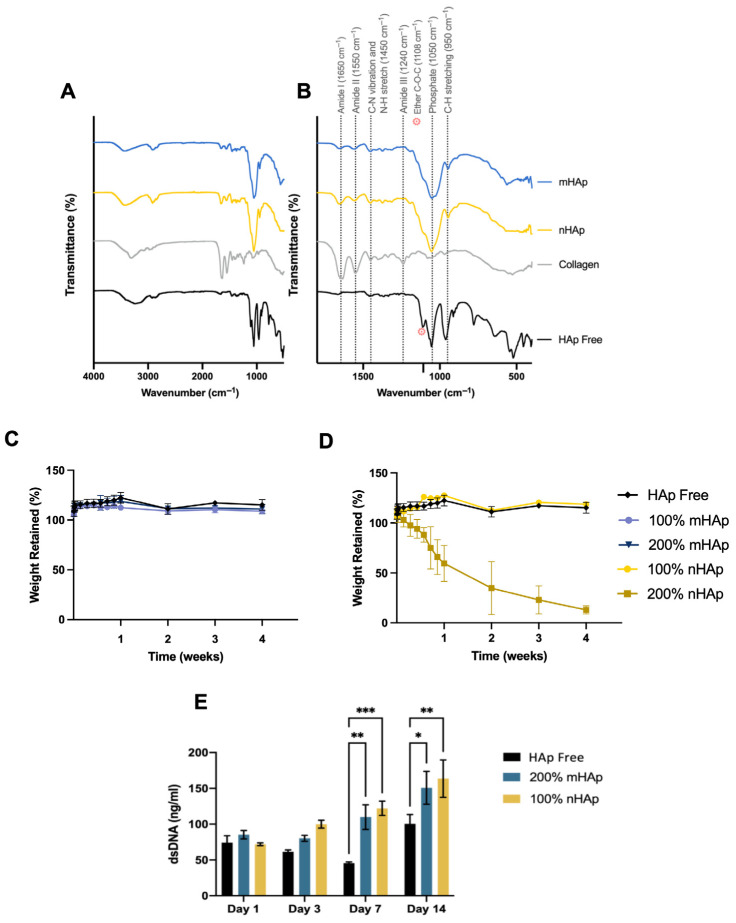
(**A**) Fourier Transform Infrared Spectroscopy spectra of lyophilised collagen, MC–collagen and MC–collagen–nHAp and mHAp samples. (**B**) Enhanced spectra showing typical bands of amides I, II, and III of collagen, phosphate banding from HAp presence, and ether peaks characteristic of glycosidic linkage in cellulose chains. Degradation resistance of gelated (**C**) micro- and (**D**) nano-hydroxyapatite hydrogels in hydrolytic environment. (**E**) Proliferation of osteoblasts cultured on thermoresponsive hydrogels. Data presented is the mean ± SD (*n* = 3). *p* ≤ 0.05 = *, *p* < 0.01 = **, *p* < 0.001 = ***.

HAp influencing collagen behaviour within hydrogel formulations was further seen with higher intensity FTIR spectra for peaks for amides I and II in Hap-containing samples, while HAp-free hydrogels lacked spectral peaks for these collagen-associated functional groups (Figure 3A,B), supporting a change in collagen and MC interaction. There are no literature reports of MC/cellulose–collagen materials separating into the observed dissociation patterns shown. There are, however, reports detailing separation of polymer and particulate phases, and this can be dependent on particulate concentrations [44]. Therefore, it is possible that HAp presence is causing a decreased interaction between collagen and HAp itself or MC. Despite the reductions in gelated stiffness, sol–gel transition temperatures were not affected by the inclusion of HAp, signifying that the HAp particles are not entirely interrupting the formation of hydrophobic interactions of methylcellulose responsible for the temperature-mediated sol–gel. Similarly, the extrusion forces through high- and low-gauge needles also were not significantly different between HAp and HAp-free formulations. However, while these extrusion experiments were completed by injecting into air, clinical application would present a degree of tissue back pressure, but the systems marketed for percutaneous vertebral injection possess needle gauges lower than our 16 G needle test [45]. Therefore, the ability to inject our developed formulations is unlikely to be impeded in scenarios with larger needle gauges.

Resistance to hydrolytic degradation was evaluated as an indicator of long-term gelated stability. Figure 3C,D show that inclusion of mHAp at 100% and 200%, as well as 100% nHAp, had no significant effect on hydrogel stability. In contrast, 200% nHAp demonstrated a marked reduction in stability from day 1, with mass retention decreasing to 97.6 ± 11.1% by day 2 and 23.1 ± 14.0% by day 21. Degradation analysis in PBS further confirmed that all formulations exhibited high stability except for 200% nHAp, which showed relatively rapid disintegration from 72 h. This loss of stability in 200% nHAp, but not in 200% mHAp, suggests that a higher fraction of nanosized particles may negatively impact long-term gel integrity. While the mechanism remains unclear, it is possible that high concentrations of nHAp either promote greater collagen fibre dissociation or interfere with hydrophobic interactions between methylcellulose chains, leading to destabilisation. Based on these findings, 200% mHAp was selected for further biocompatibility analysis due to its retained stability and high HAp content, while 100% nHAp was also chosen for its comparable stability. To assess the impact of HAp on cell viability and proliferation, dsDNA content was measured over 14 days to evaluate pre-osteoblast proliferation. Figure 3E shows that HAp inclusion enhanced cell proliferation by day 3, with significant increases observed at days 7 and 14 compared to the control. Although no significant difference was noted between mHAp and nHAp, 100% nHAp hydrogels exhibited the highest levels of proliferation and were therefore prioritised for subsequent SVT release studies. The underlying mechanism is unclear but may relate to changes in collagen fibre organisation providing additional binding sites or to the influence of HAp and its dissociation products on cell behaviour. Nevertheless, extensive evidence supports the role of HAp in improving biomaterial biocompatibility and promoting cell proliferation, particularly in hydrogel systems [46,47,48].

### 2.3. Loading and Release of Simvastatin into MC–Collagen Hydrogel Formulations

There is increasing interest in developing regenerative biomaterials that can synergistically promote bone formation and inhibit bone resorption in an effort to augment bone regeneration. Statins have been shown to have this bifunctionality. SVT has previously been shown to promote osteogenesis both in vitro and in vivo [29]. The use of statins to prevent osteoclast formation has been investigated in a small number of studies, with SVT appearing most frequently in this regard. To this end, we incorporated SVT into a HAp-free and 100% nHAp hydrogel as a therapeutic to locally alter osteogenesis and osteoclastogenesis at a site of implantation. However, due to the high water component of hydrogels, they are often unsuitable for delivery of hydrophobic molecules such as statins, as the high insolubility of these molecules can be problematic for incorporation, homogeneity, and therefore predictable release [26]. With this in mind, this study investigated the potential of the biometric thermoresponsive hydrogels as a delivery vehicle for hydrophobic statins and therapeutic efficacy of the released SVT.

**Figure 4:**
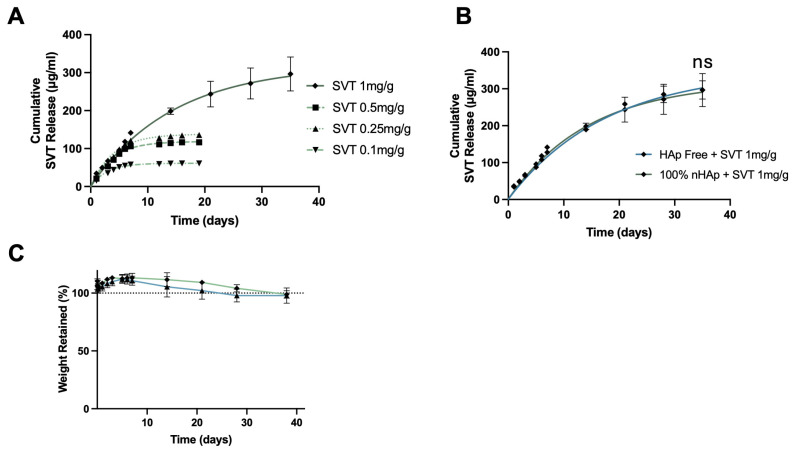
(**A**) Cumulative release of SVT from nHAp thermoresponsive hydrogels loaded with varying amounts of SVT, up to 35 days at 37 °C. (**B**) nHAp and HAp-free hydrogel formulations loaded with 1 mg/g SVT showed similar swelling and mass degradation profiles when incubated in PBS at 37 °C. (**C**) SVT cumulative release was also similar between nHAp and HAp-free formulations. Cumulative release lines plotted represent a fitted curve from non-linear regression of the mean SVT released and error bars represent the cumulative standard error *(n* = 3 for SVT 1 mg/mL and *n* = 2 for 0.1–0.5 mg/mL). A Welch two-sample *t*-test was conducted at release values on day 35, ns = not significant.

To identify a loading concentration of SVT that provided long-term sustained release, 100% nHAp hydrogels were loaded with SVT at concentrations of 1 mg, 0.5 mg, 0.25 mg, and 0.1 mg per g of hydrogel (Figure 4A). Increasing the loading concentration of SVT into the nHAp hydrogel formulations resulted in a faster rate and more sustained release profile. A 1 mg/mL loading concentration was required for sustained release over 5 weeks, with first order release profiles typical of other sustained release hydrogels, a period of fast or burst release with gradual reductions in rate, leading to plateau. Furthermore, no significant differences in the release profiles between the nHAp and the HAp-free formulations were found (Figure 4B). This kind of release profile of SVT was likely due to the physical crosslinking nature of the bulk cellulose polymer chains, conferred by the degree of methylation. The thermogelation mechanism functions as a temperature driven restructuring of polymer networks, which results in increased physical hydrophobic interaction. These hydrophobic regions could potentially act as reservoirs that facilitate adsorption of SVT. A similar strategy used by Fukuhara et al. through increasing alkyl-chain length attached to gelatin enabled increased residence and release of hydrophobic molecules [27]. Hence, the extended release profiles observed at 1 mg/g loading may result from altered interactions among the hydrophobic regions of MC during gelation. Reduced hydrophobic interactions could permit greater hydrogel swelling, leading to increased drug–polymer desorption and subsequent slow Fickian diffusion of free SVT [49]. At lower SVT loadings, this structural rearrangement may be less pronounced, potentially leaving more SVT trapped within the hydrophobic regions. There were negligible effects of loading this amount of SVT with respect to hydrolytic degradation between Hap-free and 100% nHAp formulation, as reported in Figure 4C.

**Figure A2:**
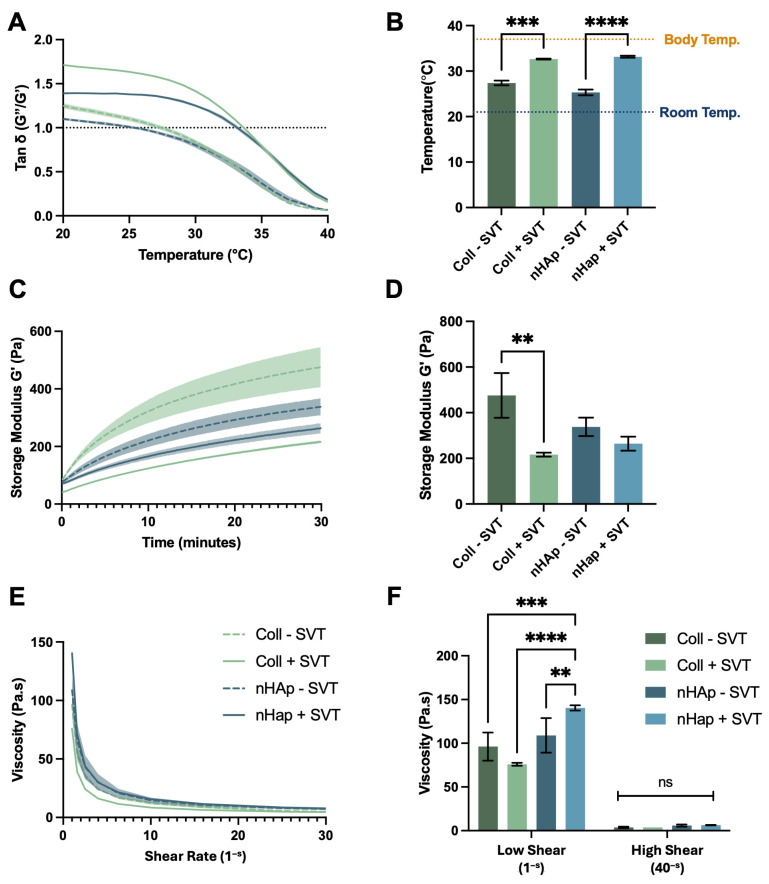
Rheological assessment of nHAp and Coll hydrogels with and without SVT loading. (**A**) Tan δ values determining sol–gel. (**B**) Sol–gel transition temperatures. (**C**) Gelation profiles achieved over 30 min incubation at 37 °C. (**D**) Maximum elastic modulus recorded during 30 min incubation at 37 °C. (**E**) Shear-thinning viscosity profiles with increasing shearing rate. (**F**) Viscosities measured at a low and high shearing rate of 1.00 s^−1^ and 40.00 s^−1^. ns = not significant *p* < 0.01 = **, *p* < 0.001 = ***, *p* < 0.0001 = ****.

SVT loading had a clear impact on the physical performance of the HAp-free and 100% nHAp formulations. Specifically, sol–gel transition temperature behaviour was significantly increased by SVT inclusion and terminal gelated stiffness was also significantly reduced (Appendix A Figure A2). While the thermoresponse did change, state transition still occurred between room temperature and body temperature, meaning characteristics for minimally invasive delivery were retained. The influence of SVT loading on the viscoelastic properties of the formulation further indicate that SVT is interacting with particular regions of MC chains that are involved in the thermogelation. While there are no reports of statins influencing the thermogelation of MC, hydrophilic and hydrophobic small molecules have been shown to influence polymer structure and in turn thermogelating behaviours, such as those in poloxamer-based hydrogels [50].

### 2.4. Osteogenic Study of SVT Release from Coll–MC Hydrogels

**Figure 5:**
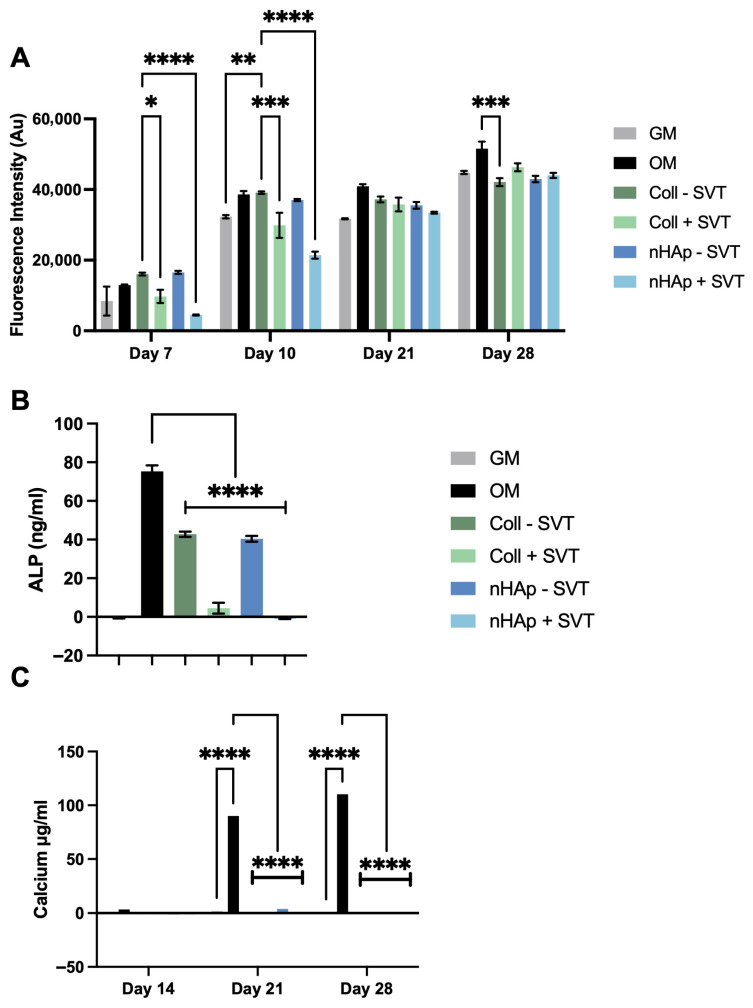
(**A**) Metabolic activity of osteoblasts upon exposure to Coll and nHAp SVT- and solvent-loaded hydrogels over 28 days. (**B**) ALP activity at day 14 osteogenic stimulation and exposure to SVT and solvent-loaded hydrogels. (**C**) Osteoblast mineralisation over 28 days osteogenic stimulation and released SVT from Coll and nHAp hydrogels. Bars represent the mean with ± SD as error bars, (*n* = 3), *p* ≤ 0.05 = *, *p* < 0.01 = **, *p* < 0.001 = ***, *p* < 0.0001 = ****.

To investigate SVT and hydrogel component release on osteogenesis, 1 mg/g SVT containing Coll and nHAp formulations were suspended over a differentiating monolayer culture. Figure 5A reports the observed metabolic activity of pre-osteoblast cells and shows that cell viability was maintained after the initial 7 days of SVT release, albeit with lower metabolic activity in Coll and nHAp + SVT exposed groups. Recovery of this differential was found at day 21, with no significant difference between all experimental groups and controls. Moreover, at day 28, equivalent metabolic activity was observed in all groups with the exception of Coll–SVT, showing a slightly lower yet statistically significant metabolic activity. Figure 5B presents ALP at day 14 as an indicator of osteogenic progress. From this, there was a clear attenuation in the production of ALP in the hydrogels compared to the control group. The inclusion of SVT in both Coll and nHAp hydrogels further inhibited the production of ALP with a significant decrease in ALP observed in both Coll and nHAp + SVT groups compared to SVT-free hydrogels. Nonetheless, no calcium production was detected in any of the hydrogel groups, with or without the inclusion of SVT, over the 28-day osteogenic culture (Figure 5C).

This data shows that, while SVT release did not reduce overall viability of pre-osteoblast cells, the presence of the SVT-loaded hydrogels seemed to inhibit osteogenesis with no ALP and calcium detected throughout the experiment, while SVT-free groups showed a delayed osteogenic response by showing ALP presence but did not show calcium production by the end of the culture period. This result contrasts early and recent evidence of SVT’s in vitro osteogenic activity in multiple stem cell and pre-osteoblast cell cultures [29,51,52]. The opposing results in this case are possibly due to the dosing of SVT. While 1 mg/g of loading was feasible and provided long-term sustained release, there is a possibility that the early burst release of SVT was overdosing the small media volumes in cell culture experiments. Despite this, a comparable SVT-releasing system loaded at 1 mg/mL showed only an approximate 20% reduction in MC3T3 proliferation while still enhancing osteogenesis [53]. Another study using a supramolecular hydrogel that released SVT through electrostatic interactions and ion exchange with laponite demonstrated that a loading dose as low as 10 μg/mL was sufficient to increase osteogenic biomarkers relative to controls [54]. In contrast, a separate study using polylactic-co-glycolic acid and biphasic ceramic scaffolds loaded with a much higher dose of SVT (10 mg/mL) reported decreases in viability of human teeth stem cells; however, it achieved osteogenic responses with this high dose [55]. These studies show contrasting effects of controlled SVT releasing systems and indicate that a lowered hydrogel SVT loading dosage might improve osteogenic response.

### 2.5. Osteoclastogenic Study of SVT Release from Coll–MC Hydrogels

**Figure 6:**
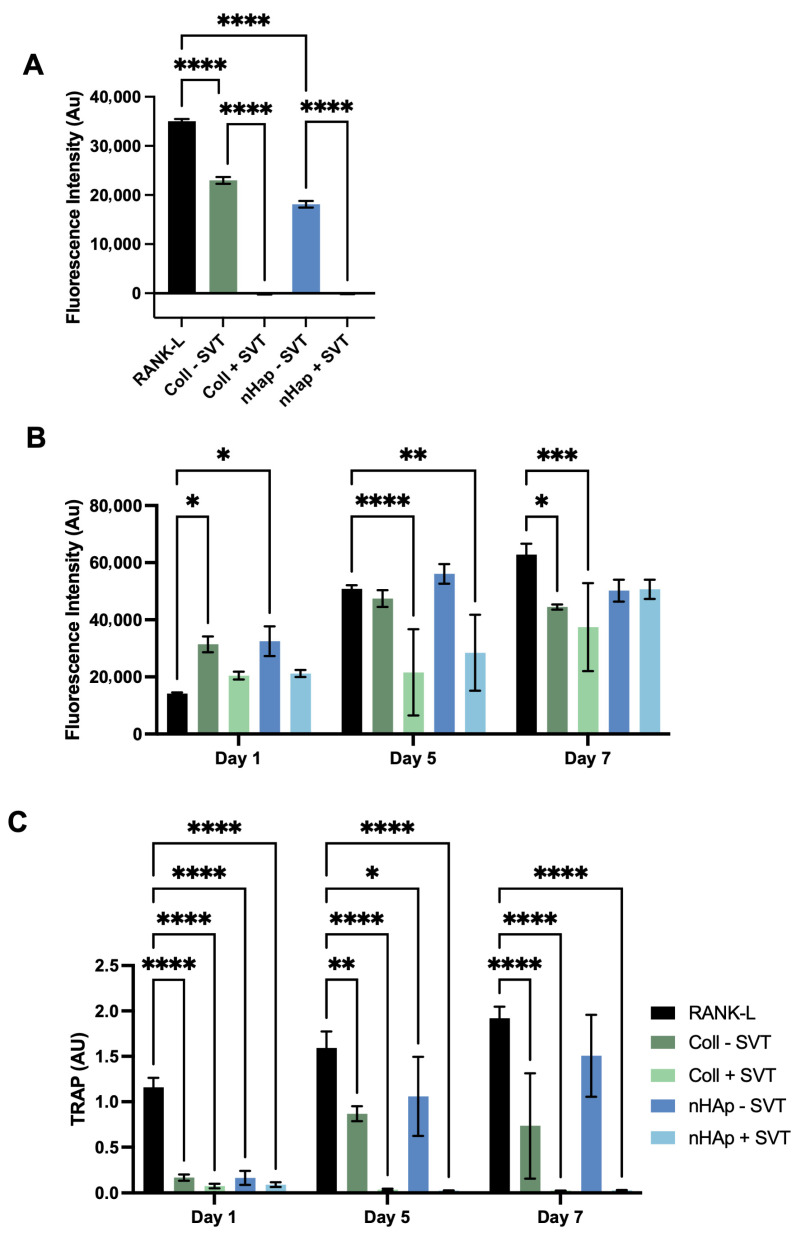
(**A**) Metabolic activity of osteoclasts cells after 3 days exposure to fresh Coll and nHAp SVT- and solvent-loaded hydrogels. (**B**) Metabolic activity of osteoclasts cells upon exposure post-burst release from Coll and nHAp SVT- and solvent-loaded hydrogels. (**C**) Quantification of RANK-L stimulated TRAP activity over 7 days stimulation in response to SVT-loaded and solvent-loaded hydrogels. Bars represent the mean with ± SD as error bars, (*n* = 3), *p* ≤ 0.05 = *, *p* < 0.01 = **, *p* < 0.001 = ***, *p* < 0.0001 = ****.

Subsequently the SVT-loaded hydrogels were tested in an osteoclastogenic culture. This perspective is important to consider with therapeutic systems that could be used for restoration of osteoporotic bone. With osteoclastogenic cultures, there was higher sensitivity to SVT release. This was seen as a loss of metabolic activity, indicating cell death, after 3 days exposure to freshly suspended SVT-loaded hydrogels, likely due to the burst release of SVT (Figure 6A). Continuing a fresh culture with the 3-day incubated hydrogels, i.e., avoiding the burst release, maintenance of viable pre-osteoclast cells over 7 days was achieved (Figure 6B). Assessing the osteoclastogenic differentiation of these cultures found no TRAP activity with SVT releasing into the culture (Figure 6C). This suggests SVT release interrupted osteoclast formation or interfered with bone resorbing function. This is largely in support of the literature that outlays the anti-osteoclastogenic mechanisms of SVT as both interrupting RANK-L signalling and reduced production of reactive oxygen species [56,57,58].

With the data indicating the requirement for dosing refinement, it is important to note that the in vitro cell culture setups employed do not reflect the complex and dynamic in vivo environment. Consequently, the release profiles and in vitro effects demonstrated may not be directly applicable within such contexts. Moreover, it is crucial to acknowledge that the crosstalk between osteoblasts and osteoclasts, a fundamental aspect of bone remodelling, was not represented in the discrete cultures employed in this study. This interplay between different cell types could significantly influence the activity of SVT on both osteoblast and osteoclast formation, potentially leading to outcomes different from those observed in this simplified culture system. Nonetheless, despite these limitations, the data presented in this study offer valuable insights into the behaviour of SVT within the hydrogel system and provide direction and knowledge for potentially efficacious drug release and improved cellular responses in simplified in vitro models.

## 3. Conclusions

In conclusion, the MC/collagen/HAp hydrogel developed in this study demonstrated suitable mechanical, thermoresponsive, and handling properties for injectable delivery, specifically, a shear-thinning viscosity (1.81 Pa.s at 100 s^−1^), a sol–gel transition temperature of 27.2 °C, a gelated elastic modulus of 345.6 Pa, and injectable extrusion forces of 45.4 N through an ultrafine needle. Furthermore, the system maintained gelated stability up to 28 days and achieved controlled release kinetics. Higher loading concentrations of 1 mg/g produced prolonged over 35 days, with the nHAp hydrogel cumulatively releasing 34.4, 67.1, 85.9, and 141.6 μg of SVT at days 1, 3, 5, and 7, respectively. This totalled a cumulative release of 296.3 μg by day 35. Functionally, the released SVT was sufficient to attenuate osteoclast differentiation observed by an absence of TRAP production; however, osteogenic enhancement was not observed under the tested dosing conditions.

This work illustrates the developed platform’s potential as a minimally invasive delivery system capable of hosting other hydrophobic drugs for therapeutic delivery to bone. Nonetheless, several limitations of the study warrant consideration, including the absence of osteoblast-osteoclast co-culture that would represent bone remodelling crosstalk and a lack of in vivo pharmacokinetic and dynamic assessment. These gaps in the study are a requirement for understanding improved dosing to achieve both osteogenesis and osteoclastogenesis. Future studies utilising SVT for sustained, local release should consider optimising loading concentrations and burst release profiles if seeking appropriate osteogenic and anti-osteoclastogenic responses.

## 4. Materials and Methods

### 4.1. Fabrication of Collagen and Methylcellulose Hydrogels

Bovine Tendon Type I Fibrillar Collagen (Collagen Solutions, Glasgow, UK) was blended in 0.5 M acetic acid at 15,000 rpm for 1.5 h using a T 25 ULTRA-TURRAX, yielding a 0.55% *w*/*v* slurry. Final 0.5% *w*/*v* collagen slurries were prepared by volume adjustment with 0.5 M acetic acid or by adding micro-hydroxyapatite (Plasma Biotal, Buxton, UK) (d(50) = 5.18 µm) or nano-hydroxyapatite (Sigma Aldrich, Arklow, Ireland) (d(50) = 200 nm) suspended in 0.5 M acetic acid. Hydroxyapatite was added at 100% and 200% *w*/*w* relative to collagen.

A 5% *w*/*w* methylcellulose dispersion was prepared by dissolving high-viscosity methylcellulose in deionised water at 70 °C, cooling under stirring, and equilibrating overnight at 4 °C.

Collagen slurries and methylcellulose dispersions were combined with deionised water and homogenised for 10 min, then equilibrated overnight at 4 °C to achieve differing concentrations of collagen content, as outlined in Table 1. Formulations without collagen used 0.5 M acetic acid instead. For subsequent collagen HAp formulations, a fixed volume of slurry with varying HAp content was added, resulting in the formulations outlined in Table 2. Resulting gels were freeze-dried at −40 °C, 200 mTorr for 35 h, cut to assist rehydration, then sterilised with ethylene oxide (EtO) for 25 h. A 5.6% *w*/*v* β-Glycerophosphate (β-GP, pH 7 adjusted with 1 M HCl) rehydration solution was sterile-filtered (0.2 µm) and added to lyophilised wafers in a volume equal to the dry mass. Rehydration occurred over 3–5 days with agitation, followed by centrifugation at 1200 g for 5 min to remove air bubbles.

### 4.2. Rheological Assessment

Hydrogel rheology was tested with a Discovery HR1 Rheometer (TA Instruments, New Castle, DE, USA) using a 40 mm, 4° cone and Peltier plate. Samples (*n* = 3) were trimmed for gap fill and conditioned at 20 °C for 120 s before measurements of gelation temperature, elasticity, and shear-thinning (parameters in Table 3).

### 4.3. Scanning Electron Microscopy (SEM)

Lyophilised samples were mounted on metal studs and sputter-coated with gold/palladium. Collagen fibril, methylcellulose, and hydroxyapatite interactions were imaged using a Zeiss Ultra Plus SEM (Oberkochen, Germany).

### 4.4. Injection Force Testing

Injectability was measured as the maximum force to extrude hydrogels at room temperature via 3 mL syringes fitted with 16 G and 26 G needles (BD). A Z050 Zwick/Roell (Worcester, UK), mechanical tester with a 5 kN load cell depressed syringes at 1 mm/s to extrude 1 mL of sample.

### 4.5. Fourier Transform Infrared Spectroscopy (FTIR)

Attenuated Total Reflection FTIR spectra of lyophilised samples were collected (400–4000 cm^−1^) using a Nicolet iS10 Smart iTX (ThermoScientific, Waltham, MA, USA).

### 4.6. Disintegration Testing

To assess gelated integrity of the formulations, 3 g of each hydrogel was centrifuged into pre-weighed 10 mL vials, thermally gelated in a 37 °C water bath, and overlaid with 3 mL prewarmed PBS. Samples were incubated at 37 °C with shaking (75 rpm). At intervals, supernatant was removed, vials weighed, and gel mass was calculated by the following formula:

((VG_x_ − V) ÷ G_0_) × 100 (1)

where VG_x_ is vial and gel mass at timepoint, V is vial mass and G_0_ is initial gel mass.

### 4.7. Simvastatin Release

Simvastatin (SVT) was loaded into hydrogels at 0.1, 0.25, 0.5, and 1 mg/g of hydrogel by slowly adding a 20 mg/mL stock (in 70% ethanol). Drug-free controls received equivalent ethanol volume. Each hydrogel was stirred to homogenise the added drug solution throughout. In total, ~0.3 mL of each formulation was applied to 4 µm PET membrane inserts in 12-well plates containing 1 mL PBS (pH 7.4), incubated at 37 °C, 5% CO_2_. At time points, inserts were removed, PBS collected and stored at −20 °C, replaced with fresh PBS, and inserts returned. SVT quantified by UV absorbance at 239 nm.

### 4.8. Cell Culture Expansion

RAW 264.7 cells were cultured in high glucose DMEM without sodium pyruvate, MC3T3-E1 cells in MEMα supplemented with 10% FBS, 1% L-glutamine, and 1% penicillin/streptomycin. Cultures were maintained at 37 °C, 5% CO_2_, media changed every 3–4 days, and passaged at ~80% confluence. For passage of cell cultures, MC3T3 cells were trypsinised and RAW 264.7 cells detached by scraping.

### 4.9. Cell Proliferation on Hydrogels

The 500 μL hydrogel formulations were pipetted into 24-well plates, centrifuged to remove air bubbles, UV sterilised for 30 min, then gelated at 37 °C for 30 min. In total, 70,000 MC3T3 cells suspended in 200 μL of media were seeded onto hydrogels and incubated 15 min at 37 °C before adding media to 1 mL total volume.

To assess cell proliferation, DNA content was assessed at days 1, 3, 7, and 14 by Quant-iT™ PicoGreen™ dsDNA Assay (ThermoScientific, Waltham, MA, USA), after lysis with 0.2 M Na_2_CO_3_ and 0.5% Triton X-100, including three freeze–thaw cycles. Fluorescence was compared to dsDNA standards diluted in lysis buffer.

### 4.10. SVT Release During Differentiation Cultures

MC3T3 cells were seeded at 4000 cells/cm^2^ and grown to 90% confluence before induction with osteogenic media (OM) containing ascorbic acid (50 μM) and β-GP (10 mM). RAW 264.7 cells were seeded at 50,000/well and stimulated with osteoclastogenic media MEMα + 10% heat-inactivated FBS and 20 ng/mL RANK-L for osteoclastogenesis.

In total, ~0.3 mL of nHAp or collagen hydrogels loaded with 1 mg/g SVT or solvent were gelated in hanging inserts for 30 min at 37 °C and placed into osteogenic and osteoclastogenic cultures.

Due to the cytotoxic effects from SVT burst release on RAW 264.7 cells observed at day 3, the well inserts were subsequently transferred to a fresh well plate with new RAW cells. This meant that sustained release effects could still be observed beyond the initial burst release of SVT.

### 4.11. Metabolic Activity Analysis

To observe viability of differentiating monolayers, 1 mL of 10% AlamarBlue (Invitrogen, ThermoScientific, Waltham, MA, USA) in DMEM was applied under hydrogel inserts for 1.5 h, and 200 μL was sampled and read at 545 nm excitation/590 nm emission using an Infinite M PLEX plate reader (Tecan, Männedorf, Switzerland).

### 4.12. TRAP Assay

To assess tartrate resistant acid phosphatase activity (TRAP), osteoclast lysates (1% Triton in sodium acetate buffer, pH 5.0) underwent 2 freeze–thaw cycles. The 100 μL samples were incubated with 100 μL TRAP assay solution (2.5 mM p-NPP, 0.1 M sodium acetate, 0.2 M KCl, 1 mM ascorbic acid, 100 μM FeCl_3_, 50 mM sodium tartrate) for 45 min at room temperature. Reaction was stopped with 50 μL 0.9 M NaOH and absorbance read at 405 nm.

### 4.13. ALP Assay

Early osteogenic alkaline phosphatase activity was assessed with SensoLyte^®^ p-NPP kits (Fremont, CA, USA). Cells were lysed using 0.2 M Na_2_CO_3_ and 0.5% Triton X-100, freeze-thawed thrice. In total, 50 μL of lysate was incubated with 50 μL of p-NPP solution for 30 min at room temperature. Reaction was stopped with 50 μL of stop solution before reading absorbance at 405 nm.

### 4.14. Calcium Assay

To test for osteogenesis, calcium content on MC3T3-E1 cells was measured by incubating monolayers in 0.5 M HCl overnight and using the Calcium LiquiColor™ (Boerne, TX, USA) test per manufacturer’s instructions, with absorbance being read at 595 nm in a Tecan plate reader.

### 4.15. Statistical Analysis

Statistical analysis was completed using Graph Pad Prism V10.4.1. One way ANOVA with Dunnett’s or Tukey’s multiple comparisons tests used to compare variable changes to a control or all groups tested, respectively.

## Figures and Tables

**Table 1:** Formulations of thermoresponsive hydrogels with varying collagen contents.

Methylcellulose	Β-Glycerophosphate	Collagen	
			0.0% *w*/*v*
		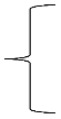	0.1% *w*/*v*
2.5% *w*/*v*	5.6% *w*/*v*		0.2% *w*/*v*
			0.3% *w*/*v*
			0.4% *w*/*v*

**Table 2:** Formulations of thermoresponsive hydrogels with varying contents of mHAp and nHAp.

Methylcellulose	Β-Glycerophosphate	Collagen	Micro/Nano-Hydroxyapatite	Abbreviation	
2.5% *w*/*v*			0.0 *w*/*v*		Hap Free
	5.6% *w*/ *v*	0.4% *w*/*v*	0.4% *w*/*v*	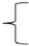	100% nHAp/ 100% mHAp
			0.8% *w*/*v*	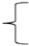	200% nHAp/ 200% mHAp

**Table 3:** Rheological tests used to assess candidate hydrogel formulations.

Experiment	Frequency	Stress	Shear Rate	Temperature	Time
Oscillatory Temperature Sweep	1 Hz	1 Pa	-	20–40 °C	1 min equilibration per 1 °C
Oscillatory Time Sweep	1 Hz	1 Pa	-	37 °C	30 min
Viscous Flow Sweep	-	-	1–500 s^−1^	21 °C	n.a

## Data Availability

The data presented in this study are available on request from the corresponding author.

## Abbreviations

The following abbreviations are used in this manuscript:

MC	Methylcellulose
Coll	Collagen
nHAp	Nano sized hydroxyapatite
mHAp	Micron sized hydroxyapatite
β-GP	Beta-Glycerophosphate
MSC	Mesenchymal Stem Cell
SVT	Simvastatin
VFF	Vertebral Fragility Fracture
HMG-CoA	3-hydroxy-3-methylglutaryl coenzyme A
SEM	Scanning Electron Microscopy
ECM	Extra Cellular Matrix
SD	Standard Deviation
FTIR	Fourier Transform Infrared Spectroscopy
PBS	Phosphate Buffer Saline
ALP	Alkaline Phosphatase
RANK	Receptor Activator Nuclear Factor Kappa
TRAP	Tartrate Resistant Acid Phosphatase
PET	Polyester
UV	Ultraviolet
DMEM	Dulbecco’s Modified Eagle Medium
FBS	Foetal Bovine Serum
OM	Osteogenic Media
MMP	Matrix Metallo Protease
PEG	Poly Ethylene Glycol
RANKL	Receptor Activator of Nuclear Factor Kappa B Ligand
MAPK	Mitogen-Activated Protein Kinase
AKT	Protein Kinase B
